# Characterization of Two New *brown midrib1* Mutations From an EMS-Mutagenic Maize Population for Lignocellulosic Biomass Utilization

**DOI:** 10.3389/fpls.2020.594798

**Published:** 2020-11-16

**Authors:** Wangdan Xiong, Yu Li, Zhenying Wu, Lichao Ma, Yuchen Liu, Li Qin, Jisheng Liu, Zhubing Hu, Siyi Guo, Juan Sun, Guofeng Yang, Maofeng Chai, Chunyi Zhang, Xiaoduo Lu, Chunxiang Fu

**Affiliations:** ^1^Key Laboratory of Biofuels, Shandong Provincial Key Laboratory of Energy Genetics, Qingdao Institute of Bioenergy and Bioprocess Technology, Chinese Academy of Sciences, Qingdao, China; ^2^Grassland Agri-Husbandry Research Center, College of Grassland Science, Qingdao Agricultural University, Qingdao, China; ^3^University of Chinese Academy of Sciences, Beijing, China; ^4^Institute of Molecular Breeding for Maize, Qilu Normal University, Jinan, China; ^5^Collaborative Innovation Center of Crop Stress Biology, Henan Province and Institute of Plant Stress Biology, Henan University, Kaifeng, China; ^6^Biotechnology Research Institute, Chinese Academy of Agricultural Sciences, Beijing, China

**Keywords:** cinnamyl alcohol dehydrogenase, EMS mutagenesis, biofuels, C4 grass, forage

## Abstract

Gene mutations linked to lignin biosynthesis are responsible for the *brown midrib (bm)* phenotypes. The *bm* mutants have a brown-reddish midrib associated with changes in lignin content and composition. Maize *bm1* is caused by a mutation of the cinnamyl alcohol dehydrogenase gene *ZmCAD2*. Here, we generated two new *bm1* mutant alleles (*bm1-E1* and *bm1-E2*) through EMS mutagenesis, which contained a single nucleotide mutation (Zm*cad2-1* and Zm*cad2-2*). The corresponding proteins, ZmCAD2-1 and ZmCAD2-2 were modified with Cys103Ser and Gly185Asp, which resulted in no enzymatic activity *in vitro*. Sequence alignment showed that CAD proteins have high similarity across plants and that Cys103 and Gly185 are conserved in higher plants. The lack of enzymatic activity when Cys103 was replaced for other amino acids indicates that Cys103 is required for its enzyme activity. Enzymatic activity of proteins encoded by *CAD* genes in *bm1-E* plants is 23–98% lower than in the wild type, which leads to lower lignin content and different lignin composition. The *bm1-E* mutants have higher saccharification efficiency in maize and could therefore provide new and promising breeding resources in the future.

## Introduction

Lignin is a phenolic polymer that provides mechanical support to plant organs and protects the plant from pathogen attacks, thus playing a vital role in plant growth and development (Bhuiyan et al., 2009; Barros et al., 2015; Gallego-Giraldo et al., 2020). Lignin is produced from the polymerization of monolignols, which are synthesized through the phenylpropanoid pathway. Lignin polymer consists of three predominant types: *p*-hydroxyphenyl (H), guaiacyl (G) and syringyl (S) subunits (Vanholme et al., 2010). The presence of lignin in the plant cell wall reduces (CWRs) the efficiency of ethanol production from lignocellulosic biomass and the digestibility of forage for animal consumption (Chen and Dixon, 2007). Thus, modifying lignin content in plants can be an effective approach for forage improvement (Jung and Ni, 1998).

Lignin content and its composition are particularly important in plants. Studying the monolignol metabolic pathways is key to develop successful genetic breeding programs (Dixon and Barros, 2019). Brown midrib (*bm*) mutants are a good model to study such metabolic pathways. They have a reddish midrib phenotype and variation in lignin content. This phenotype was initially described about 90 years ago and was discovered in C_4_ grasses (i.e., maize, sorghum, and pearl millet) (Jorgenson, 1931; Sattler et al., 2010). Six *bm* mutants (*bm1*-*6*) have been isolated in maize. Mutation loci have been analyzed in *bm1*-*5* (Vignols et al., 1995; Halpin et al., 1998; Tang et al., 2014; Li et al., 2015; Xiong et al., 2019). Genes responsible for *bm1* and *bm3* encode for cinnamyl alcohol dehydrogenase (CAD) and caffeic acid *O*-methyltransferase (COMT), respectively (Vignols et al., 1995; Halpin et al., 1998). Mutated genes in both *bm2* and *bm4* are involved in one-carbon metabolism, and encode for functional methylenetetrahydrofolate reductase (MTHFR) and folypolyglutamate synthase (FPGS) respectively (Tang et al., 2014; Li et al., 2015). The recently cloned gene in *bm5* encodes for 4-coumarate: coenzyme A ligase (4CL) (Xiong et al., 2019). Besides changes in lignin content and composition, *bm* mutants show improved forage efficiency (Vignols et al., 1995; Godin et al., 2016), which makes them promising breeding resources.

CAD catalyzes the NADPH-dependent reduction of hydroxycinnamyl aldehydes to their alcohol derivatives which are then incorporated into lignin (Goffner et al., 1992; Vanholme et al., 2010). The *bm1* maize mutant was caused by a transposon insertion in the first intron of *CAD2* (*GRMZM5G844562*) gene (Chen et al., 2012). Decreased CAD activity in *bm1* reduces its Klason lignin content and G and S monomers yield (Halpin et al., 1998). The *bmr6* phenotype is the result of a mutation in the *CAD* gene in sorghum, same as in maize *bm1* (Saballos et al., 2009; Sattler et al., 2009). The *bmr6* mutant also exhibits altered lignin content and composition with higher saccharification efficiency (Sattler et al., 2009). CAD catalyzes the final and essential step in the monolignol biosynthesis and genetic modification in the *CAD* gene leads to changes in lignin content and composition (Yan et al., 2019). CAD deficiency decreases overall lignin content, alters lignin structure and increases enzymatic recovery of sugars in rice, tomato, *Brachypodium*, and switchgrass (Saathoff et al., 2011; d’Yvoire et al., 2013; Li et al., 2019; Martin et al., 2019).

In this study, two new maize *bm1* mutant alleles were obtained through ethyl methanesulfonate (EMS) mutagenesis. To study the impacts of the mutation on ZmCAD2 function, the expression level of *ZmCAD2* was determined in *bm1-E* mutants and Z58. Quantitative RT-PCR (qRT-PCR) was used to determine whether the missense mutations have impact on *ZmCAD2* gene expression. Unlike previous reported maize *bm1* mutants, the new *bm1* alleles resulted from a single base mutation and the missense mutation caused the encoded proteins lost activity. The study indicated the conserved amino acids Cys103 and Gly185 were important sites for CAD2 activity and it provided new alleles impacting lignin biosynthesis in maize, as well as cell wall digestibility efficiency and plant breeding.

## Materials and Methods

### Plant Material

Maize plants were grown in the greenhouse at 26°C, 16 h of light and 8 h of darkness. Maize *bm1* stock (*bm1-PI228174*) was derived from the Maize Genetics COOP Stock Center. The *bm1-E* mutants (*bm1-E1* and *bm1-E2*) were obtained from an EMS mutagenic population which was generated from the inbred line Z58^1^. Phenotypic identification was confirmed through hybrid complementation tests with *bm1* plants. Z58 plants with no reddish midrib phenotype were used as the wild type control.

### Gene Cloning and Expression Analysis

Genomic DNA was extracted from leaf tissue using the 2 × CTAB protocol (Porebski et al., 1997). To determine the mutations responsible for the *bm1* phenotype, the *ZmCAD2* gene was cloned and sequenced using the primers listed in Supplementary Table S1. The Zm*CAD2* genes were amplified from the mutants, sequenced and aligned against the Z58 genome.

Total RNA was extracted from the midrib of 60 days-old plant using Trizol according to the manufacturer’s instructions (Invitrogen, United States). The extracted RNA was treated with DNaseI. The expression level of the *CAD* gene in the midrib was analyzed using quantitative reverse transcription polymerase chain reaction (qRT-PCR) assay (Tang et al., 2014).

### Expression and Purification of Recombinant Proteins

The *CAD* coding region was amplified from Z58 and *bm1*-*E* plants and then sub-cloned into pET32a vector. The recombinant vectors were transferred into BL21 *Escherichia coli* cells. Cells were cultured at 37°C in Luria-Bertani medium. Isopropyl β-D-thiogalactopyranoside (IPTG) was added to a final concentration of 0.3 mM at mid-log phase (A600 = 0.4∼0.6). Cells were continued to be incubated at 18°C for 16 h and harvested by centrifugation at 5,000 × *g* for 5 min. Soluble proteins were extracted by sonication and the recombinant proteins were purified with Ni affinity column (GE Healthcare) for further enzyme activity (Youn et al., 2006).

### Enzyme Activity and Protein Structure Analyses

Enzymatic activity was determined as described by Scully et al. (2016). Midribs of the second to fifth leaves from the top were collected from 60-day old *bm1-E* mutants and Z58 wild type plants. Powdered fresh midribs (∼0.5 g) were extracted for 3 h at 4°C in protein extraction buffer (Liu et al., 2012). The samples were centrifuged at 13,000 × *g* for 20 min at 4°C, and the extracts were desalted on PD-10 columns (Pharmacia) and used for CAD enzyme activity assay. The assay was performed using 200 ng of purified recombinant CAD proteins or 100 μg of total protein for midrib extracts with different substrates. The reaction was carried out at 30°C for 30 min in 300 μL of 100 mM Tris-HCl (pH 7.5), 0.4 mM NADP, 1 mM DTT, 5% ethylene glycol, and 0.14 mM substrates (*p*-coumaraldehyde, coniferaldehyde, and sinapaldehyde).

The three-dimensional structure of CAD in *bm1*-*E* mutants was predicted using the SWISS-MODEL^2^. SWISS-MODEL generated models by homology using amino acid sequences to infer the stoichiometry and the overall structure of the assembly (Bertoni et al., 2017).

### Chemical Analysis

Maize midribs were collected from the fourth-to-fifth leaves of 60-day-old plants. CWRs were prepared and used for further chemical analysis as described previously (Chen and Dixon, 2007). Lignin content was quantified using the acetyl bromide method. The thioacidolysis method was used to measure the lignin composition (Lapierre et al., 1986; Hatfield et al., 1999). For enzymatic hydrolysis analysis, CWR was digested by direct exposure to a cellulase and cellobiase mixture for 72 h (as untreated samples). Enzymatic saccharification of samples was performed following the analytical procedure of the National Renewable Energy Laboratory (LAP-009). Saccharification efficiency was calculated as the ratio of sugars released by enzymatic hydrolysis versus sugar content in the CWR. Sugar release was analyzed using the phenol-sulfuric acid assay method (Dubois et al., 1956). Cellulose and hemicellulose were extracted as described in Yu et al. (2014). Monomeric sugars were determined by HPLC (Agilent 1200 Series LC system with 1200 Series refractive index detector) equipped with an Aminex HPX-87P column (Agilent Technologies).

### Statistical Analysis

Three biological replicates were used for all collected data and the mean values were used for statistical analyses. Data from each trait were subjected to Student’s *t*-test. One or two asterisks indicate significance corresponding to *P* < 0.05 or 0.01. Standard errors were provided in all tables and figures as appropriate.

## Results

### Identification of New *bm1* Alleles

**FIGURE 1:**
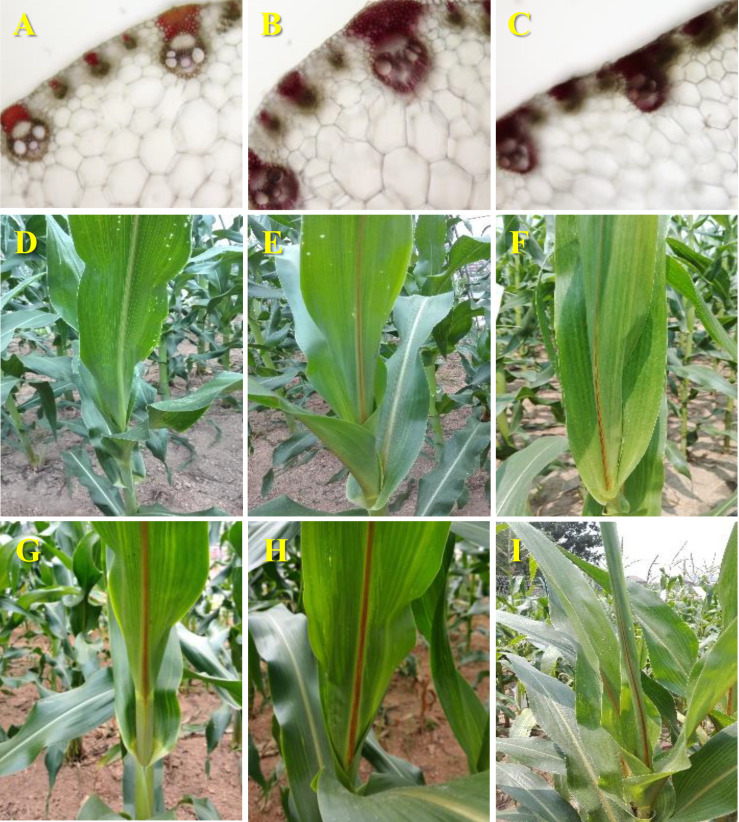
Morphological characterization of the *bm1-E* mutants. Midribcross sections stained with acid phloroglucinolof the fourth leaf collected from 60-days old wild-type Z58 **(A)**, *bm1-E1* **(B)**, and *bm1-E2* **(C)**. Adaxial view of themidrib of 45-days old wild-type Z58 **(D)**, *bm1-E1* **(E)**, *bm1-E2* **(F)**, *bm1* **(G)**, hybrid *bm1* × *bm1-E1* **(H)**, and hybrid *bm1* × *bm1-E2* **(I)**. Cross sections and adaxial view of the midribs from three independent materials of each plants were used for histochemical assay and phenotypic measurements.

Two putative *bm* mutants were isolated from the EMS-mutagenic population. Stained midrib cross sections with phloroglucinol-HCl showed a darker brown in these two new *bm* mutants than in Z58. This indicates higher accumulation of aldehyde derivatives in their lignified tissues and the incorporation of cinnamyl aldehydes, the substrates for CAD (Figures 1A–C). Indeed, the allelism tests with the previously described maize *bm1* mutant (Gene stock ID: *bm1*-PI267186) confirmed that two new EMS-mutagenized *bm* mutants were *bm1* alleles (*bm1-E*) (Figures 1D–I).

**FIGURE 2:**
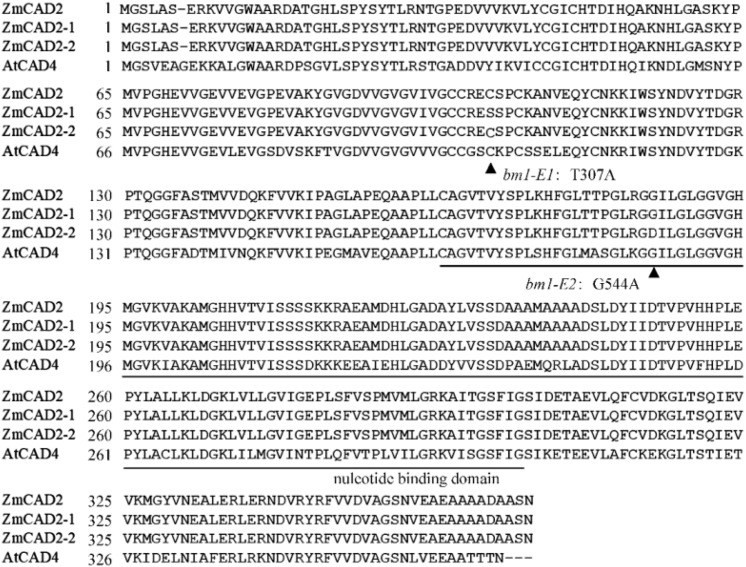
Amino acid sequence alignment of predicted cinnamyl alcohol dehydrogenase proteins. The mutant amino acid was highlighted in red color. The protein sequences of ZmCAD2 was aligned with ZmCAD2-1, ZmCAD2-2, and AtCAD4 proteins using ClustalW. AtCAD4: *Arabidopsis thaliana* P48523.1; ZmCAD2: *Zea maize* GRMZM5G844562. ▲ indicates the mutant amino acids; the line indicates the nucleotide biding domain.

In *bm1*-*E* mutants, two transition missense mutations (T357A and G554A) were identified in the *ZmCAD2* coding region (Figure 2). In *bm1-E1* plants, Cys103 was converted into Ser, while Gly185 was converted into Asp in *bm1-E2* plants (Figure 2).

### Impacts of the Mutation on ZmCAD2 Function

**FIGURE 3:**
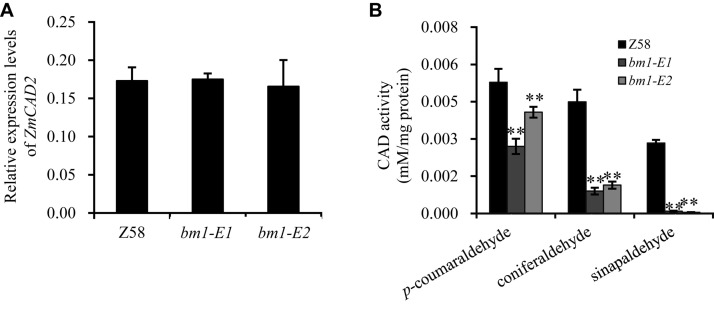
Effects of *ZmCAD2* mutation in gene expression and CAD enzymatic activity in midribs of the fourth leaf collected from 60-days old plants. **(A)** Gene expression levels of *ZmCAD2*. **(B)** CAD enzymatic activity in different substrates: *p*-coumaraldehyde, coniferaldehyde, and sinapaldehyde. Values shown are means ± SD from three technical replicates and three biological replicates. Two asterisks indicate significance corresponding to *P* < 0.01 (Student’s *t*-test).

To study the impacts of the mutation on ZmCAD2 function, the expression level of *ZmCAD2* were determined in *bm1-E* mutants and Z58. No significant differences were detected in the expression levels of *ZmCAD2* between the wild type and two *bm1-E* mutants (Figure 3A). Although the missense mutation did not affect the expression of the *ZmCAD2* gene in *bm1-E* plants, its impact in the enzymatic activity remains uncertain. The recombinant corresponding mutation proteins of *Zmcad2* of *bm1-E* (ZmCAD2-1 and ZmCAD2-2) were expressed *in vitro*. The point mutations Cys103Ser and Gly185Asp both resulted in no CAD activity compared with the wild type (Table 1 and Supplementary Figure S1).

**TABLE 1:** Enzymatic activity of ZmCAD2 and its mutated versions in different substrates *in vitro*.

Name	Amino acids	Substrates		
		*p*-Coumaraldehyde	Coniferaldehyde	Sinapaldehyde
Z58	Cys103Gly185	√	√	√
*bm1-E1*	Ser103Gly185	ND	ND	ND
*bm1-E2*	Cys103Asp185	ND	ND	ND
M1	His103Gly185	ND	ND	ND
M2	Lys103Gly185	ND	ND	ND
M3	Gly103Gly185	ND	ND	ND
M4	Asp103Gly185	ND	ND	ND
M5	Met103Gly185	ND	ND	ND

*M: mutation;*

*ND: no enzymatic activity detected.*

*In vivo* assays showed that CAD enzymatic activity significantly decreased in the midrib of two *bm1-E* mutants (Figure 3B). In the *p*-coumaraldehyde substrate, CAD activity in *bm1-E1* and *bm1-E2* was 51.1 and 71.1% lower than in the wild type. In coniferaldehyde, it was only 20.0 and 25.4% compared to the wild type (Figure 3B). In sinapalaldehyde, the enzymatic activity of *bm1-E1* and *bm1-E2* was reduced to 3.3 and 1.6% of the wild type (Figure 3B).

### Impacts of *ZmCAD2* Point Mutations on Predicted Protein Structure

CAD belongs to the medium chain dehydrogenase/reductase (MDR) superfamily. It is composed of two distinct domains: the nucleotide-binding domain and the catalytic domain (Figure 2) (Jornvall and Hoog, 1995). Here, a mutation in Cys103Ser observed in ZmCAD2-1 protein led to no CAD activity (Table 1 and Supplementary Figure S1). The predicted protein structure showed that Cys103Ser would not change the three-dimensional structure of ZmCAD2 (Supplementary Figure S2). When Cys103 was replaced with any other amino acid (His, Asp, Lys, Gly, and Met), there was no enzymatic activity using *p*-coumaraldehyde, coniferaldehyde, and sinapalaldehyde as substrates (Table 1 and Supplementary Figure S2). Therefore, our results suggest that Cys103 is essential for CAD catalytic activity.

The nucleotide-binding domain of ZmCAD2 is located in the 163–301 region (Figure 2). The Gly185Asp mutation in ZmCAD2-2 protein did not change ZmCAD2 three-dimensional protein structure except for the amino acid substitution (Supplementary Figure S2). Gly and Asp were different in structure, as Asp contained a side-chain. A replacement of Gly185 for Asp resulted in the lack of CAD enzymatic activity for ZmCAD2-2. This is consistent with former observations in sorghum (Scully et al., 2016). In *bmr6-23* and *bmr6-1103*, Gly184 was mutated into Asp, and no CAD enzyme activity was detected (Scully et al., 2016).

### Impact of *bm1-E* Mutant on Lignin Content and Composition

**FIGURE 4:**
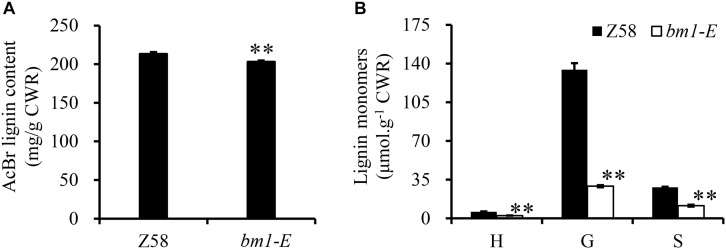
Effect of *bm1-E* mutation on lignin content **(A)** and lignin composition **(B)**. Midribs of the second to fifth leaves from the top were collected from 60-days old *bm1-E* mutant and Z58 wild type maize. Values are means ± SE from three biological replicates. Two asterisks indicate significance corresponding to *P* < 0.01 (Student’s *t*-test).

Reduction of CAD activity in plants would affect the biosynthesis of lignin. Herein, the lignin content and its composition were analyzed in Z58 and *bm1-E* plants. Lignin content in *bm1-E* mutants decreased by 5.11 and 4.38% compared to Z58 (Figure 4A). Furthermore, levels of H, G, and S lignin were also lower than in the wild types (reduced to 43.3, 21.7, and 40.9%, respectively) (Figure 4B).

### *bm1-E* Biomass Has Increased Glucose Yields Following Enzymatic Saccharification

**FIGURE 5:**
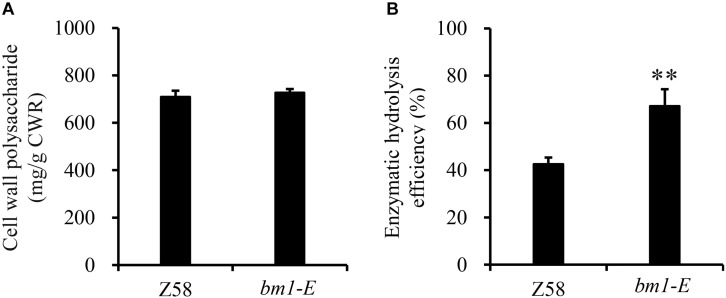
Effect of *bm1-E* mutation on total sugar content **(A)** and enzymatic hydrolysis efficiency **(B)**. Stalk samples were collected from 90-days old *bm1-E* mutants and Z58 wild type plants. Values are means ± SE from three biological replicates. Two asterisks indicate significance corresponding to *P* < 0.01 (Student’s *t*-test).

To determine the impact of *bm1-E* mutant on fermentable sugars, the cellulose and hemicellulose contents were measured with CWRs. The cellulose content in *bm1-E* mutants was 6.02% higher (346.9 mg/g CWR) than in the wild type Z58 (327.2 mg/g CWR) (Table 2). The hemicellulose content was also changed in *bm1-E* plants. Xylose and arabinose contents were lower in *bm1-E*, while glucose, galactose, rhamnose, and galacturonic acid levels were higher (Table 2). The xylose content accounts for 73.4% of the total hemicellulose in the wild type Z58, and in *bm1-E* mutant was reduced from 256.7 to 200.8 mg/g CWR (i.e., a relative decrease of 21.8%) (Table 2). The total cell wall polysaccharide was not significantly changed in *bm1-E* plants (Figure 5A). Additionally, *bm1-E* mutants showed an enzymatic saccharification efficiency of 67.1% at 72 h against 42.5% in Z58 plants (Figure 5B).

**TABLE 2:** Cellulose content and monosaccharide composition (mg/g CWR) of non-cellulosic cell wall carbohydrates in Z58 and *bm1-E* plants.

Plant	Cellulose	Xylose	Arabinose	Glucose	Mannose	Galactose	Rhamnose	Galacturonic acid
Z58	327.2 ± 6.42	256.7 ± 22.65	28.8 ± 1.68	44.3 ± 2.46	5.5 ± 2.20	5.1 ± 0.29	1.8 ± 0.09	7.4 ± 0.26
*bm1-E*	346.9 ± 5.88**	200.8 ± 12.92**	23.1 ± 2.39**	56.8 ± 2.03**	4.7 ± 1.08	6.5 ± 0.72**	2.4 ± 0.47**	8.2 ± 2.11**

*Midrib samples were collected from 60-days old Z58 and *bm1-E1* plants.*

*Values are means ± SE from three biological replicates.*

*One or two asterisks indicate significance corresponding to *P* < 0.05 or 0.01 (Student’s *t*-test).*

## Discussion

The *ZmCAD2* gene was first identified and mapped in maize *bm1* mutants in Halpin et al. (1998). Mutations in this gene dramatically reduce its transcription rate and enzymatic activity, which results in reduced lignin content and changes in lignin composition (Halpin et al., 1998). In this study, two new maize *bm* mutants (*bm1-E1* and *bm1-E2*) were obtained through EMS mutagenesis and were certified to be *bm1* alleles. The expression level of *ZmCAD2* did not significantly changed in *bm1-E* in comparison to the wild type Z58 (Figure 3A). Both ZmCAD2-1 and ZmCAD2-2 showed no enzymatic activity *in vitro* (Table 1). The missense mutations in *bm1-E* reduced CAD activity by 75–98% in the midribs of *bm1-E* plants, especially in coniferaldehyde and sinapaldehyde (Figure 3B). CAD catalyzes the final step in the monolignol biosynthesis. A mutation in the *CAD* gene leads to the accumulation of hydroxycinnamyl aldehyde derivatives in maize and *Medicago truncatula* (Halpin et al., 1998; Zhao et al., 2013). The accumulation of cinnamyl aldehydes in the midrib was confirmed with phloroglucinol-HCl staining (Halpin et al., 1998). The *bm1-E* mutants exhibited a decrease in lignin content and altered lignin composition due to changes in the phenylpropanoid metabolic pathways (Figure 4).

Total lignin content in *bm1-E* was 4.7% lower, while it was significantly reduced by 20% in former identified maize *bm1* (Halpin et al., 1998). There are two possible explanations for this: one is that different methods were adopted to extract and analyze the lignin content. The other reason is that maize stalk was examined instead of the midrib. Although the lignin content did not vary, lignin composition was significantly reduced for H, G, and S lignin in *bm1-E* plants.

Conserved CAD proteins are important for its enzymatic activity. They have two main domains: the nucleotide-binding domain and the catalytic domain (Figure 2). Cys103 and Gly185 are generally conserved in CAD proteins (Youn et al., 2006; Scully et al., 2016). Cys103 is in the catalytic domain in ZmCAD2, which is an important function in the MDR family (Youn et al., 2006). ZmCAD2-1 protein displayed no enzymatic activity, and no activity was detected when Cys103 was replaced for other amino acids (Table 1). When Gly185 was changed for Asp, ZmCAD2 also lost its enzymatic activity (Table 1). Previous study has suggested that a point mutation Gly185Asp in OsCAD2 protein sequence of rice *gh2* results in complete loss of the CAD and SAD activities which leads to impaired lignin biosynthesis and a reddish-brown pigmentation in the hull and internode in rice (Zhang et al., 2006). The same point mutation Gly185Asp was observed in the ZmCAD2 of *bm1-E2*, implying that Gly185 is crucial for CAD structure and function. Interestingly, another point mutation Gly184Asp in CAD sequence of sorghum *Bmr6-23 and -1103* mutants can lead to nearly undetectable bmr6 protein *in vitro* (Scully et al., 2016). It was formerly suggested that changing Gly for Asp without a side-chain would impact hydrophilicity, and that Gly185 occurred in close to residues predicted for dimerization, which would interfere the enzyme forming a homodimer (Scully et al., 2016). In contrast, the changing Gly185 for Asp in OsCAD2 only affects CAD activity rather than gh2 protein expression *in vitro* (Zhang et al., 2006). Thus, the conserved amino acids in CAD may play different roles in its enzymatic structure and function.

The Cys103 and Asp185 mutations resulted in loss of its enzyme activities (Table 1). In spite of no enzymatic activity in the mutant ZmCAD2 proteins, other CAD proteins compensated the cinnamyl alcohol dehydrogenase activity. Still, CAD enzyme activities were dramatically reduced *in vivo*, especially in coniferaldehyde and sinapaldehyde (Figure 3). CAD enzymatic activity in *bm1-E* was 75.6–80% lower than the wild type in coniferaldehyde, and 96.7–98.4% in sinapalaldehyde (Figure 3B). The OsCAD2 protein sequences in *gh2* (Zhefu802 as the background), undergoing a same point mutation with ZmCAD2 in *bm1-E*2, lost its function (Zhang et al., 2006). And *gh2* plant obviously exhibited reddish-brown in the internode (Zhang et al., 2006). The CAD activities for total *gh2* plant proteins in all tested tissues (except for midrib) were dramatically reduced using coniferaldehyde as substrate, while its activity was not detectable using *gh2* proteins from panicle, hull, sheath, internode, and root tissues using sinapylaldehyde as substrate (Zhang et al., 2006). It was speculated that CAD isoenzymes unevenly exist in maize different tissues and less CAD homologs using sinapylaldehyde as substrate exists in maize midrib. Still, the enzyme activity reduction seems not be consistent the reduced S lignin content. The main reason for this is that the thioacidolysis method (Figure 3B) mainly proceeds by the cleavage of β-*O*-4 aryl ether lignin structures which account for about 50% of all lignin linkages in maize.

Lignin abundance and structure are correlated to saccharification efficiency in plants, which could improve the saccharification efficiency of cell wall polysaccharides (Sattler et al., 2010). Indeed, cell wall saccharification efficiency of polysaccharides was higher in *bm1-E* plants. Three main reasons could explain it: (i) The lignin content is reduced in *bm1-E* (Figure 4A). Lignin is a major obstacle to cell wall degradation, and its content is negatively correlated with biomass enzymatic saccharification efficiency (Tarasov et al., 2018). (ii) S/G ratio was increased in *bm1-E* mutant compared with Z58 wild type (Figure 4B). Recent articles have indicated that the S/G ratio may play dual roles in biomass enzymatic hydrolysis for sugar yields (Studer et al., 2011; Yoo et al., 2017; Alam et al., 2019). (iii) The arabinose and xylose level of hemicellulose was significantly reduced in *bm1-E* mutant (Table 2). It has been proposed that the major arabinose should be associated with lignin for cell wall network construction, which somehow explains the reduced arabinose level in both mutants should somewhat disassociate with lignin inter-linkage, resulting in raised biomass porosity for high enzymatic hydrolysis (Wang et al., 2016). *ZmCAD2* is the major gene responsible for lignin biosynthesis in maize. *CAD2* mutants have a brown midrib phenotype, and variable lignin accumulation. The missense mutation in the ZmCAD2 in *bm1-E* showed no enzyme activity *in vitro*, and CAD enzymatic activity was severely affected *in vivo* (Figure 3B and Table 1). However, catalysis of the hydroxycinnamyl aldehydes to their corresponding alcohols derivatives was still observed (Figure 4). It is possible that other *CAD* genes were responsible for this catalytic activity. *CAD* genes belong to the MDR family and has many homologous in plants (Youn et al., 2006). There are nine *CAD*-like putative genes in *Arabidopsis*, of which AtCAD4 and AtCAD5 show the highest catalytic activity and are mainly responsible for lignin formation *in vivo* (Sibout et al., 2005; Youn et al., 2006). Twelve *CAD*-like genes have been previously identified in rice and *OsCAD2* was the major *CAD* gene responsible for lignin biosynthesis in rice (Zhang et al., 2006; Hirano et al., 2012). Interestingly, *AtCAD4*, *AtCAD5*, *OsCAD2*, *Bmr6*, and ZmCAD2 were clustered in a clade together (Hirano et al., 2012). This indicates that *CAD* gene function is conserved across different plant groups, and that *ZmCAD2* is the main gene responsible for CAD catalytic ability in maize.

Like other *bm* mutants in maize, the saccharification efficiency was improved in *bm1-E* mutants (Chen and Dixon, 2007). The new *bm1* mutations presented here would provide more resources for research study, lignocellulosic biomass utilization, and plant breeding.

## Data Availability Statement

The raw data supporting the conclusions of this article will be made available by the authors, without undue reservation.

## Author Contributions

CF, XL, and WX conducted experimental design and manuscript writing. WX, YL, ZW, LM, YCL, LQ, JL, JS, GY, and MC conducted in experiments and data collection. CF, YL, WX, ZH, SG, and CZ analyzed the data. All authors approved the final manuscript.

## Conflict of Interest

The authors declare that the research was conducted in the absence of any commercial or financial relationships that could be construed as a potential conflict of interest. The handling editor declared a shared affiliation, though no other collaboration, with several of the authors WX, YL, ZW, LM, YCL, and CF.

## Footnotes

1 http://www.elabcaas.cn/memd/

2 https://swissmodel.expasy.org/
