# Supplementary material for: Characterization of Two New brown midrib1 Mutations From an EMS-Mutagenic Maize Population for Lignocellulosic Biomass Utilization
Source: Front Plant Sci. 2020 Nov 16;11:594798. doi: 10.3389/fpls.2020.594798 (PMC7703671; doi:10.3389/fpls.2020.594798)
Supplement: Supplementary file 1 [file Data_Sheet_1.docx]

**Supplementary data**

**Figure S1.** The CAD activity of purified recombinant ZmCAD2, ZmCAD2-1 and ZmCAD2-2 proteins.

**Figure S2.** Disruptions to *bm1-E* protein structure caused by Cys103Ser (A) and Gly185Asp (B) point mutations.

**Table S1.** Primers used in the study.

**Figure S1.** The CAD activity of purified recombinant ZmCAD2, ZmCAD2-1 and ZmCAD2-2 proteins.

**Figure S2.** Disruptions to *bm1-E* protein structure caused by Cys103Ser (A) and Gly185Asp (B) point mutations.

The impact of the point mutations on ZmCAD2 structure was modeled using SWISS-MODEL with the X-ray structure of a cinnamyl alcohol dehydrogenase (PDB ID: 2cf5). In panel A, the mutant Cys103Ser ZmCAD2 protein is represented as a red ribbon overlaid on the wild type protein (yellow ribbon). In panel B, the protein structure of the Gly185Asp mutant protein (red and yellow) is overlaid over the wild type CAD2 protein (blue and purple).

**Table S1.** Primers used in the study.

| Name | Forward primer sequences (5'-3') | Reverse primer sequences (5'-3') |
| --- | --- | --- |
| ZmCAD2-full length | TCCCGAATCGAATGGGGAGCCTGGC | GACGCGGTGCCATCAGTTGCTGGCC |
| ZmCAD2-qRT | GGTACGTGAACGAGGCGCTGG | GCACGCAGACTCGACCCGAACG |
| ZmCAD2-pET32a | GCTGATATCGGATCCGAATTCATGGGGAGCCTGGCGT | TTGTCGACGGAGCTCGAATTCTCAGTTGCTGGCCGCA |
| ZmCAD2-Cys103His | CGCGAGCACAGCCCCTGCAAG | CTTGCAGGGGCTGTGCTCGCG |
| ZmCAD2-Cys103Lys | CGCGAGAAGAGCCCCTGCAAG | CTTGCAGGGGCTCTTCTCGCG |
| ZmCAD2-Cys103Gly | CGCGAGGGCAGCCCCTGCAAG | CTTGCAGGGGCTGCCCTCGCG |
| ZmCAD2-Cys103Asp | CGCGAGGACAGCCCCTGCAAG | CTTGCAGGGGCTGTCCTCGCG |
| ZmCAD2-Cys103Met | CGCGAGATGAGCCCCTGCAAG | CTTGCAGGGGCTCATCTCGCG |
